# Age-period cohort analysis of AIDS incidence rates in Rio de Janeiro, Brazil, 1985–2009

**DOI:** 10.1186/1478-7954-11-22

**Published:** 2013-11-26

**Authors:** Cristina Pinheiro Nádia Rodrigues

**Affiliations:** 1 Departamento de Tecnologias da Informação e Educação em Saúde/ Universidade do Estado do Rio de Janeiro (UERJ), Rio de Janeiro, Brazil; 2 Escola Nacional de Saúde Pública (ENSP)/Fundação Oswaldo Cruz (FIOCRUZ), Rio de Janeiro, Brazil

**Keywords:** Acquired immunodeficiency syndrome, Sexually transmitted diseases, Age effect, Cohort effect, Period effect, Epidemiology

## Abstract

**Introduction:**

The long average incubation time from HIV infection to AIDS makes it difficult to estimate the recent tendencies of HIV from AIDS incidence data. The objective of this study was to investigate the effects of three temporal components in AIDS incidence in the state of Rio de Janeiro, Brazil - age, period, and cohort.

**Methods:**

Age-specific AIDS incidence rates per 100,000 from Rio de Janeiro (Brazil) were calculated for both sexes using five-year age classes from 1985 to 2009 based on reported data from the Notifiable Disease Information System of the Brazilian Ministry of Health and from census population counts. Multivariate negative binomial models were used to analyze the risk of AIDS by age, period, and birth cohort.

**Results:**

From 1985 to 2009, AIDS incidence initially increased with age in each birth cohort and then decreased (except for individuals born from 1971–1979 to 1986–1994). High peaks in the rates in each birth cohort were detected in 1995–1999 for males and in 2000–2004 for females. Multivariate analysis showed the maximum risk of AIDS in the 30–34 age group and 1958–1962 birth cohort.

**Conclusion:**

Age, birth cohort, and period effects all may have influenced the AIDS incidence rates over the period investigated. From 1985 to 1999, comparison of the tendencies (by age) of the period with the birth cohort revealed opposing tendencies in individuals older than 29 years and in the youngest age groups (0 to 14 years). From 2000 to 2009, a strong age effect can be observed in both sexes. Consistent changes in period tendency curves suggest the occurrence of period effects. A reduction in the intensity of the risk of AIDS can be observed after 2000–2004.

## Introduction

The past three decades have seen great biomedical and behavioral advances in preventing, diagnosing, and treating human immunodeficiency virus (HIV) infection [1,2]. The treatment coverage of antiretroviral therapy has expanded. Just over 8 million people in low- and middle-income countries were receiving treatment in 2011, with coverage reaching 54% (range 50–60%). It is estimated that 15 million people may be receiving treatment by 2015 [3]. Since 1999, the number of new infections has fallen by 19%. New HIV infections are declining in many of the countries most affected by the epidemic. AIDS incidence fell by more than 25% between 2001 and 2009 in 33 countries, 22 of them in sub-Saharan Africa, the region most affected by the AIDS epidemic [4]. New HIV infections in sub-Saharan Africa appear to have peaked in the late 1990s, and HIV prevalence seems to have declined slightly, although it remains at an extremely high level of 5% among adults [5]. On the other hand, in Scandinavian countries, adult prevalence of AIDS in 2009 was around 0.1% [6]. Contrary to the overall trend, in seven countries, five of them in Eastern Europe and Central Asia, AIDS incidence increased by more than 25% between 2001 and 2009 [7]. A study conducted in 28 countries of the European Union and European Economic Area revealed that the number of diagnosed HIV infections has been holding relatively stable, from 6.5 per 100,000 individuals in 2004 to 5.7 per 100,000 individuals in 2010. Since 2004, more than 27,000 new HIV infections have been diagnosed and reported each year, resulting in a cumulative total number of over 370,000 HIV infections reported since the beginning of epidemic [8].

At the start of the AIDS epidemic, most of those who became infected with HIV were homosexual males and users of injected drugs. Although this pattern has changed over time, several regions and countries do not fit the overall global trend of AIDS. There is evidence of a resurgence of HIV in several high-income countries among men who have sex with men [9]. In Eastern Europe and Central Asia, high rates of HIV transmission continue to occur in networks of people [7]. The results of an age-period-cohort study conducted in Europe from 1981 to 1994 to investigate AIDS incidence rates among injecting drug users showed that the epidemic changed from low to intermediate levels in Austria, Greece, and the northwestern European countries from the 1965–1969 to 1960–1964 cohorts and from low to high levels in France, Italy, and Switzerland. Spain showed high incidence among recent birth cohorts, while in Portugal, the epidemic was at an early and expanding phase [10].

According to a study carried out in the United States, the proportion of women with AIDS increased significantly between 1982 and 1986 from 12% to 26%. The authors agree that this trend may prove to be a good marker for following trends in heterosexual transmission [11]. In 1995, women accounted for 19% of AIDS cases in adults; AIDS rates per 100,000 women were highest in heterosexual contacts (5.5) and among women living in metropolitan areas with more than 1 million residents (15.9). The greatest increase in AIDS incidence rate was observed in heterosexually infected women born between 1970 and 1974, i.e., women who were 14 to 18 years old in 1988 [12].

The advances of interventions in last decades have significantly reduced perinatal transmission of AIDS [13]. Despite this fact, the number of women living with HIV is an essential contributing factor to the increase of childhood AIDS cases related to perinatal transmission from the mother [11]. According to a UNAIDS report, the total number of children born with HIV globally has decreased with increasing access to services to prevent mother-to-child transmission of HIV. In 2009, it was estimated that 370,000 children (220,000 – 520,000) had contracted HIV during the perinatal and breastfeeding period. This number fell to 500,000 (320,000 – 670,000) in 2001 [7]. New HIV infections among children in high-income countries have already been virtually eliminated. The number of new infections among children fell by 93% between 1992 and 2005 in the US [5].

The number of annual AIDS-related deaths worldwide is steadily decreasing from the peak of 2.1 million (1.9 million–2.3 million) in 2004 to an estimated 1.8 million (1.6 million–2.1 million) in 2009. This decline reflects the increased availability of antiretroviral therapy, as well as care and support to people living with HIV, particularly in middle- and low-income countries [7].

### AIDS in Brazil

Since the AIDS epidemic began in Brazil, 608,230 cases have been reported (January 1980 - June 2011), of which 65.4% occurred in males and 34.6% in females. These percentages have been changing over time, with the differences between genders narrowing [14]. In the last decade, the AIDS incidence rate in Brazil has stabilized at levels still considered high by the Brazilian Health Ministry. In 2008, it reached 18.2 per 100,000 inhabitants, while in the same period, the incidence of AIDS in Western Europe did not exceed 2 per 100,000 inhabitants [6,15]. Data from the Health Ministry show that AIDS increased gradually until 2002, then began to decline gradually until 2007, after which it started to grow again. In 2009, there was an increase of 2.9% compared to the prior year [14].

Fitting the global trend, most of the people that became infected with HIV in Brazil in the beginning of AIDS epidemic were homosexual males, injecting drug users, and hemophiliacs. This pattern has changed since 1993, when more cases of AIDS were attributed to heterosexual rather than homosexual transmission. From 1995 until 2007, the share of injecting drug use as the exposure category in the occurrence of new AIDS cases declined from 27.5% to around 10% among adult males and 21.9% to just over 4% among adult females. With the implementation in 1988 of HIV blood-screening tests, HIV transmission through blood transfusion and blood products began to decline, and today HIV transmission resulting from contaminated blood products and blood transfusions can be considered practically non-existent [15].

Over time, the Brazilian population has gone through a transition process of the AIDS epidemic, which changed from the first pattern rooted among active homosexual men to a new one, which increasingly affects women [16]. Although men still account for the majority of infections, women represent an increasing share of the epidemic. The ratio of male-to-female AIDS cases decreased over time, from 15-to-1 in 1986 to 1.5-to-1 in 2008 [14].

Since the introduction of antiretroviral therapy in Brazil, the AIDS mortality rate significantly declined, from 9.6 annual deaths per 100,000 inhabitants in 1996 to 6.1 in 2008 [15]. The number of cases in which HIV was transmitted from pregnant mothers to their children also declined after the availability of antiretroviral treatment and interruption of breastfeeding. The rate of vertical transmission in the state of São Paulo was 2.7% (1.86 - 3.94, 95% confidence interval) in 2006, which represents a decrease of 83.1% compared with the 1988–1993 period [17].

The objective of this study was to investigate the effects of three temporal components in AIDS incidence in the state of Rio de Janeiro – age, period, and cohort – with the aim of shedding light on the observed trends in recent years.

## Materials and methods

Information of reported cases of AIDS of residents of Rio de Janeiro by sex, age, and year of diagnosis recorded over the 25-year period from January 1, 1985 to December 31, 2009 was obtained from the Notifiable Disease Information System (SINAN/DATASUS) of the Brazilian Ministry of Health. Population data from the Brazilian censuses of 1980, 1991, 2000, and 2010 and estimates for between-census years, stratified by age and sex, were used to calculate the incidence rates. AIDS incidence for each calendar year of the study was obtained by summing all notified cases of residents and dividing by the person-year for the respective year. Incidence rates per 100,000 inhabitants were calculated for both sexes using five-year age classes to investigate trends by period and by birth cohort. Period and birth cohort of five calendar years were considered; the central year was used as label: 1987 (1985–89), 1992 (1990–94), 1997 (1995–99), 2002 (2000–04), and 2007 (2005–09) were middle-points of periods; 1925 (1923–27), 1930 (1928–32), 1935 (1933–37), 1940 (1938–42), 1945 (1943–47), 1950 (1948–52), 1955 (1953–57), 1960 (1958–62), 1965 (1963–67), 1970 (1968–72), 1975 (1973–77), 1980 (1978–82), 1985 (1983–87), 1990 (1988–92), 1995 (1993–97), and 2000 (1998–2002) were middle-points of birth cohorts. Period and birth-cohort effects also were investigated graphically using curves constructed separately for each sex [18-22]. Multivariate negative binomial models were used to analyze the effects of age, period, and birth cohort on AIDS risk. Poisson regression is the most popular distribution for modeling counts, although it provides a poor fit in overdispersion situations [23]. Due to overdispersion, multivariable negative binomial regression models were used in this study. The negative binomial model is employed as a functional form that relaxes the overdispersion restriction of the Poisson model. In this study, two models were constructed to explain AIDS occurrence. The first model included the explanatory variables “age group,” “sex,” and “period”, while the second included the variables “birth cohort,” “sex,” and “period”. In both models a logarithmic link function (logarithm of the population) used as an offset was added.

Model specifications:

$$Y\sim \mathrm{BN}\;\left(µ,\theta \right)$$

1st model: log (μ_cases_) = α + β_1_ * sex + β_2_ * age-group + β_3_ * period + log (population)

2nd model: log (μ_cases_) = α + β_1_ * sex + β_2_ * birth-cohort + β_3_ * period + log (population)

In this study, age group, period, and birth cohort were included into the models as dummy variables, using as reference categories the 0–4 age group, 1985–90 period, and 1940 birth cohort, respectively.

Graphs and analyzes were performed using software R 3.0.0 and Microsoft Excel (Microsoft Office 2013).

## Results

**Figure 1:**
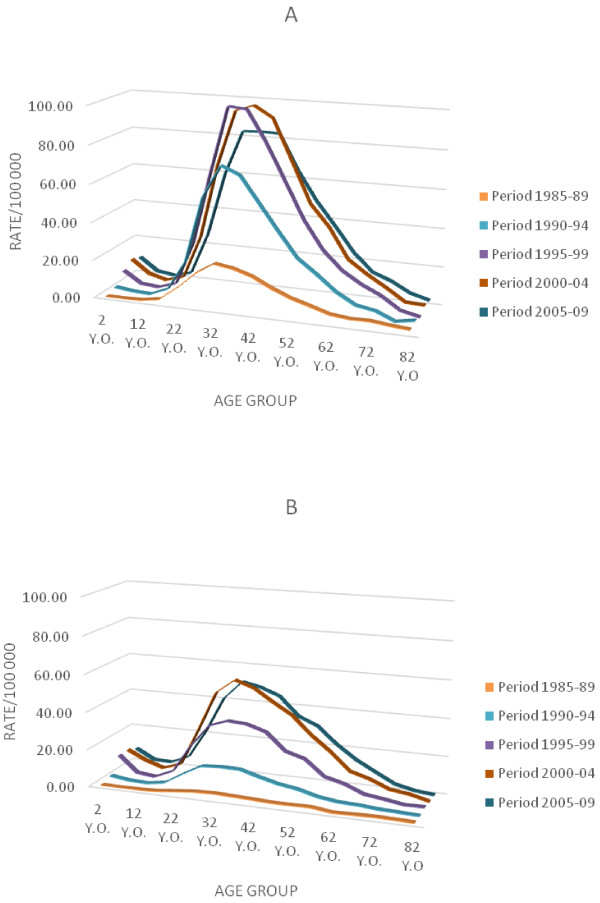
**Graph showing data from time-series study of AIDS incidence by age from 1985 to 2009 in Rio de Janeiro state.** The values are derivable from Table 1. The solid lines represent the midpoints of the periods. **A**: Male incidence. **B**: Female incidence.

Table 1 and Figure 1 show the incidence rates of AIDS in Rio de Janeiro by age in each period. The results were plotted at the middle points of five-year age groups from time-series study (1985 to 2009; middle points: 1987, 1992, and 1997) in males and females. One can observe a decreasing AIDS trend with age in the youngest age groups (aged 0 to 14) in each period (except for males in 1985–89). This trend changes from 10–14 to 30–34 years of age, where it starts to increase with age. For individuals from 30–34 to 70–74 years of age, there is a consistent trend of decreasing incidence of AIDS with age in each of these periods.

**Table 1:** Data from a series of cross-sectional studies of AIDS incidence (per 100,000) in males and females, from Rio de Janeiro, by age and survey data (calendar time)

**Age group**	**Periods (midpoint)**									
	***Incidence rates per 100,000***									
	**1987**		**1992**		**1997**		**2002**		**2007**	
***Years***	***Males***	***Females***	***Males***	***Females***	***Males***	***Females***	***Males***	***Females***	***Males***	***Females***
**0 - 4**	0.03	0.14	1.90	1.96	7.91	10.23	11.29	10.19	9.65	8.08
**5 - 9**	0.00	0.00	0.71	0.56	1.80	1.59	4.28	5.41	2.87	2.78
**10 - 14**	0.33	0.07	0.28	0.13	0.94	0.48	1.80	2.14	1.44	2.38
**15 - 19**	1.96	0.44	4.25	1.68	4.04	4.73	4.73	5.54	3.93	6.67
**20 - 24**	9.25	1.54	19.87	7.59	26.65	19.76	27.64	25.66	26.49	22.19
**25 - 29**	17.88	2.52	54.64	12.97	64.33	31.69	66.95	47.01	59.96	41.26
**30 - 34**	23.89	2.90	71.53	13.62	99.44	35.21	95.57	54.51	83.17	51.16
**35 - 39**	22.39	2.51	67.35	13.43	98.51	34.46	98.80	51.07	83.56	48.98
**40 - 44**	19.35	2.11	53.97	10.51	82.63	31.29	92.99	44.47	83.17	45.07
**45 - 49**	14.48	1.59	40.60	7.94	63.69	22.16	72.02	38.42	64.86	35.21
**50 - 54**	10.46	1.58	27.82	6.33	44.57	19.15	50.62	28.81	49.71	30.93
**55 - 59**	7.59	1.71	20.61	3.58	30.16	10.91	39.32	20.95	37.41	22.08
**60 - 64**	4.54	0.29	12.68	2.14	20.64	8.28	23.66	11.71	24.47	14.93
**65 - 69**	3.48	0.52	7.08	1.73	14.54	4.00	16.79	8.75	15.09	9.37
**70 - 74**	3.65	0.76	5.35	0.84	9.90	2.59	10.88	4.40	11.31	4.05
**75 - 79**	2.54	0.55	1.12	0.48	3.41	0.89	4.46	3.03	6.02	1.64
**≥ 80**	1.70	0.29	2.90	0.00	1.24	1.06	4.04	0.47	3.38	0.59

**Figure 2:**
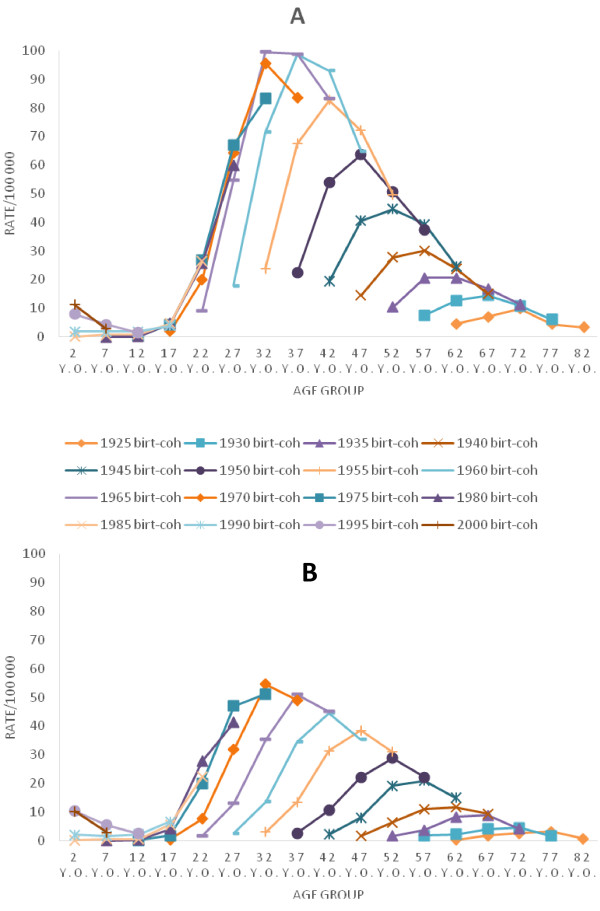
**Data from temporal series study conducted from 1985 to 2009, in Rio de Janeiro state.** The solid lines represent the midpoints of birth cohorts (from 1923 to 2002). **A**: Males AIDS incidence rates, by age. **B**: Females AIDS incidence rates, by age.

Figure 2 displays the birth cohort data from 1985 to 2009 using a different approach. In this new approach the solid lines connect points by birth cohort (rather than by period as in the first figure). Each of these solid lines represents one birth cohort. A U-shaped age pattern can be observed in the youngest age group (younger than 22 (20–24) years old). This indicates that for each birth cohort, AIDS incidence tends initially to decrease with increasing age and then increases. For people from 22 (20–24) years old, an inverted U-shape age pattern can be seen; that is, for each birth cohort, AIDS incidence tends initially to increase with increasing age and then decreases. For everyone older than 32 (30–34) years, there is a peak in the rates in 1997 (1995–99) in each male birth cohort and in 2002 (2000–04) in each female birth cohort.

**Figure 3:**
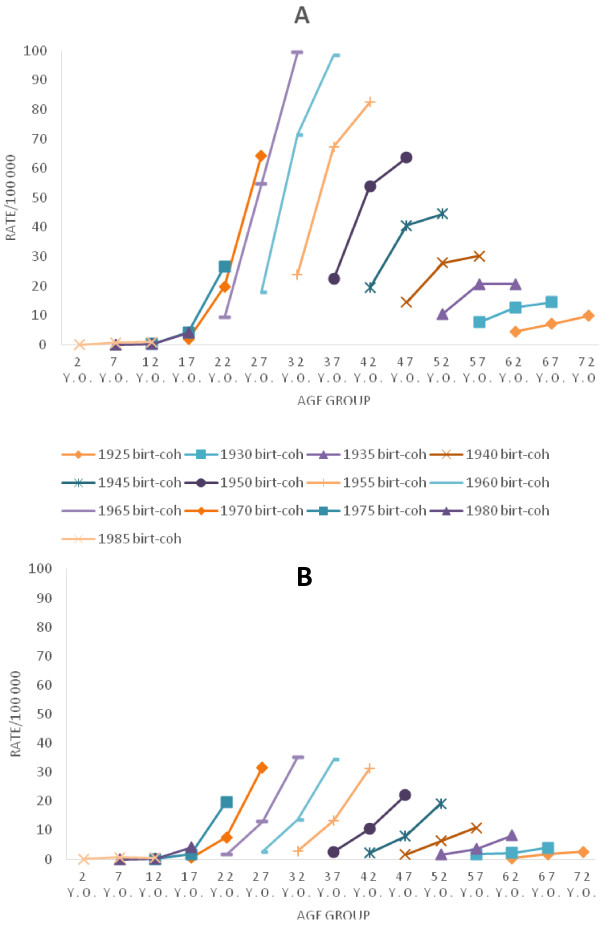
**Data from temporal series study conducted from 1985 to 1999, in Rio de Janeiro state.** The solid lines represent the midpoints of birth cohorts (from 1923 to 1989). **A**: Males AIDS incidence rates, by age. **B**: Females AIDS incidence rates, by age.

Figure 3 displays the birth cohort data from 1985 to 1999. In contrast to the age tendency by period observed in the first graph, Figure 3 shows a growing risk of AIDS with increasing age for individuals born between 1923 and 1989.

**Figure 4:**
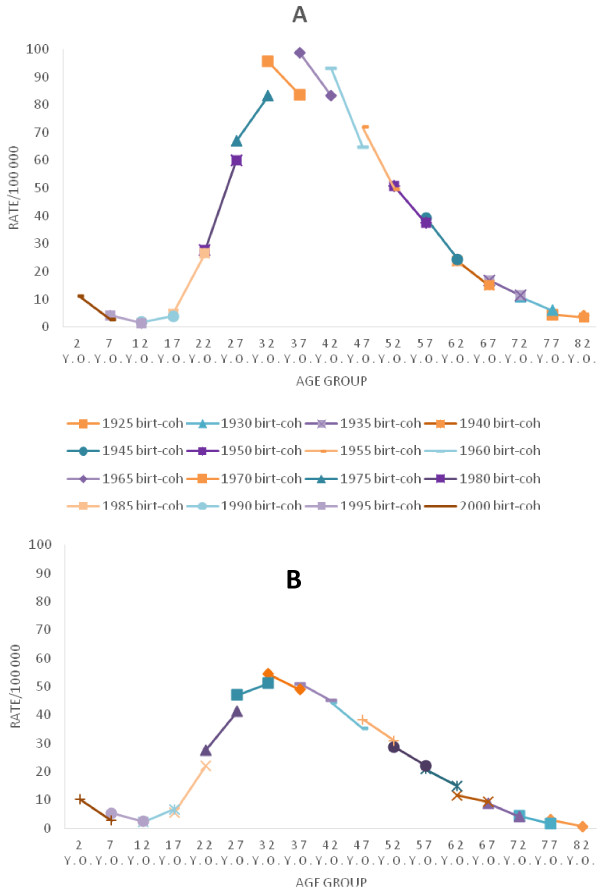
**Data from temporal series study conducted from 2000 to 2009, in Rio de Janeiro state.** The solid lines represent the midpoints of birth cohorts (from 1923 to 2002). **A**: Males AIDS incidence rates, by age. **B**: Females AIDS incidence rates, by age.

Figure 4 shows the same approach presented in Figure 3 for the last years analyzed, 2000 to 2009. Decreasing incidence rates with age can be observed for individuals born after 1990 (1988–92 birth cohort) and before 1975 (1973–77 birth cohort), while there is an increasing tendency with age for individuals born from 1975 (1973–77) to 1990 (1988–92). Also, one can observe that successive birth cohort pairs, for both male and female age groups, are practically overlapping over this period.

**Figure 5:**
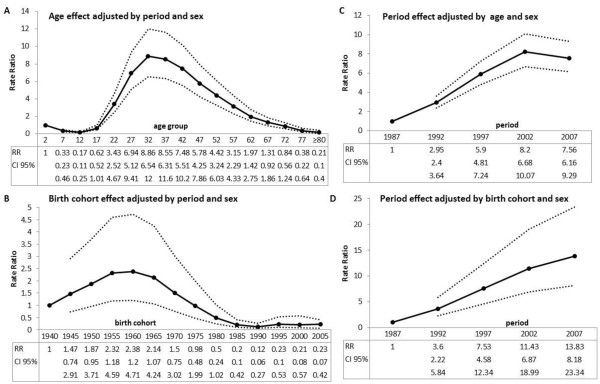
**Adjusted rate ratios of AIDS by age, period and birth cohort**^**1**^**, Rio de Janeiro State (Brazil), 1985–2009. **^1^In both cases were used negative binomial models, response variable “AIDS cases” and offset “logarithm of the population”. Models **A** and **C** included explanatory variables: “age group”, “sex” and “period”. Models **B** and **D** included explanatory variables: “birth cohort”, “sex” and “period. Dotted lines represent upper and lower boundaries of the 95% confidence interval. Midpoints were used to represent five units” intervals of age, period and cohort.

Table 2 and Figure 5 show the effect of age, period, and birth cohort for AIDS risk using a multivariate analysis approach. The rate ratio of AIDS between men and women, adjusted for age and period, was 2.26. Figure 5A shows the age effect of AIDS adjusted by period and sex. A peak in the rate ratios in individuals aged 30–34 years is evident. From the ages of 14–19 to 30–44, the rate ratio of AIDS incidence increases approximately 52 times (ranging from 0.17 to 8.86). Thereafter, the rate ratio begins to decrease. Figure 5B shows the birth cohort effect adjusted by period and sex. There is a peak in the rate ratios in the 1960 (1958–62) birth cohort. For individuals born from 1940 (1938–42) to 1960 (1958–62), the rate ratio of AIDS increases approximately twice. For individuals born from 1960 (1958–62) to 1990 (1988–92) the rate ratio decreases approximately 20 times (ranging from 2.38 to 0.12). For those born after 1990, the rate ratio remains constant. Figure 5C shows the period effect adjusted by age and sex. There is a peak in the rate ratios in 2002 (2000–04). From 1987 (1985–89) to 2002 (2000–04), the rate ratio of AIDS increased approximately eight times. After this time, the rate ratio began to decrease. In Figure 5D, period effect is again analyzed, but now the rate ratios are adjusted by birth cohort and sex. Again, one can observe an increasing tendency from 1987, but it remains increasing after 2002 (2000–04). From 1987 (1985–89) to 2007 (2005–09), the ratio rate of AIDS increases approximately 14 times.

**Table 2:** **Adjusted coefficients of AIDS by sex, age, period, and birth cohort**^**1**^**, Rio de Janeiro, 1985-2009**

**Factors**	**Categories**	**Coefficient**	**CI 95%**		**P-value**
Sex^1^	Male	0.82	0.7	0.94	<0.001
Reference: fem					
Age group^2^	5-9	-1.11	-1.47	-0.78	<0.001
(Years)	10-14	-1.77	-2.21	-1.39	<0.001
Reference: 0-4	15-19	-0.48	-0.65	0.01	0.06
	20-24	1.23	0.92	1.54	<0.001
	25-29	1.94	1.63	2.24	<0.001
	30-34	2.18	1.88	2.49	<0.001
	35-39	2.15	1.84	2.45	<0.001
	40-44	2.01	1.71	2.32	<0.001
	45-49	1.75	1.45	2.06	<0.001
	50-54	1.49	1.18	1.8	<0.001
	55-59	1.15	0.83	1.47	<0.001
	60-64	0.68	0.35	1.01	<0.001
	65-69	0.27	-0.08	0.62	0.14
	70-74	-0.17	-0.58	0.22	0.38
	75-79	-0.97	-1.51	-0.45	<0.001
	≥80	-1.56	-2.3	-0.92	<0.001
Birth cohort^3^	1945	0.39	0.39	-0.3	0.27
(Midpoint)	1950	0.63	0.63	-0.05	0.07
Reference-1940	1955	0.84	0.84	0.17	0.01
	1960	0.87	0.87	0.18	0.01
	1965	0.76	0.76	0.07	0.02
	1970	0.41	0.41	-0.29	0.23
	1975	-0.02	-0.02	-0.73	0.95
	1980	-0.69	-0.69	-1.43	0.04
	1985	-1.61	-1.61	-2.3	<0.001
	1990	-2.12	-2.12	-2.81	<0.001
	1995	-1.47	-1.47	-2.3	<0.001
	2000	-1.56	-1.56	-2.53	<0.001
	2005	-1.47	-1.47	-2.66	0.02
Period^2^	1992	1.08	0.88	1.29	<0.001
(Midpoint)	1997	1.77	1.57	1.98	<0.001
Reference: 1987	2002	2.1	1.9	2.31	<0.001
	2007	2.02	1.82	2.23	<0.001
Period^3^	1992	1.28	0.8	1.76	<0.001
(Midpoint)	1997	2.02	1.52	2.51	<0.001
Reference: 1987	2002	2.44	1.93	2.94	<0.001
	2007	2.63	2.1	3.15	<0.001

^1^Two negative binomial models have been used to assess the effect of the factors of sex, age, period, and cohort on the risk of AIDS occurrence.

^2^Explanatory variables of the first model: “age group,” “sex,” and “period”.

^3^Explanatory variables of the second model: “birth cohort,” “sex,” and “period”.

## Discussion

This study revealed that gender, age, birth cohort, and period effects are important factors for explaining AIDS incidence rates in Brazil.

The risk of AIDS in men, adjusted by age and period, was approximately two times greater than in women. Comparing the incidence rates of AIDS among sexes, it was found that although males show higher rates than females, women had the greatest increase during the period investigated. The largest differences occurred in older individuals aged 60–64 years and in young individuals aged 10–19 years. For example, for individuals aged 10–14, while in males the incidence increased 4.36 times from 1987 (1985–89) to 2007 (2005–09), in females it increased 34 times in the same period. The greatest difference occurred in the 60-64-year-old age group. In this age group, while male incidence increased 5.4 times from 1987 (1985–89) to 2007 (2005–09), in women it increased 51.5 times over the same period. The results of a study that covered the Brazilian population of a northeastern state from 1985 to 1998 also detected that incidence rates showed an annual growth that was higher in females than in males. The relative risk of the disease was about 1.2 for men, 1.3 for women, and 1.22 for both [24].

Looking at each calendar year curve (Figure 1), one can get the impression that between 30 and 39 years old is the age group at greatest risk of developing AIDS. But that is not the case; the people who make up the 30–34 and 35–39 age groups in the periods investigated experienced a rising risk of AIDS in early life. To demonstrate this, a different approach was used in this study to analyze the same data: lines were plotted to connect the points by birth cohort (rather than by period as in the first figure). Each of these lines represents one birth cohort (Figures 2,3,4). These results indicate that incidence of AIDS is strongly determined by year of birth. Especially for individuals older than 29 years in 1985–99 (Figure 3), the results indicate that younger cohorts are at higher risk of AIDS compared to older generations (e.g., for men aged 40–44 years, the risk of AIDS per 100,000 inhabitants was 19.35 for those born in 1945 (1943–47), 53.97 for those born in 1950 (1948–52), and 82.63 for those born in 1955 (1953–57), while for women in the same age group, the risks were 2.11, 10.51, and 31.29 for those born in 1945, 1950, and 1955, respectively). This rising cohort trend is also observed after the data were adjusted by period and sex for individuals born from 1940 to 1960. For those born after this time period, a decreasing trend of the rates can be observed (Figure 5B). One factor that may have contributed to the decline in trend from the 1958–62 cohort is the growing use of condoms. Brazilian data by birth cohort show accelerated growth in condom use at first intercourse, especially for individuals born between 1969 and 1973 [25].

Increasing trends of AIDS by age (adjusted by period and sex) were observed in individuals between 10–14 and 30–34 years old. In the other age groups, a decreasing trend of AIDS risk by age was observed (Figure 5A). Verdecchia et al. (1994) investigated the effects of age, period, and cohort in the Italian population in the 1980s and 1990s, and found that the age at highest risk of infection was 25 years in men and 23 years in women [26]. Besides the socio-cultural differences between the Brazilian and Italian population, the present study is more current (1985–2009) and this might be why the age at highest risk for AIDS has been found to be older, 30–34 years old.

From 1985 to 1999, comparing the tendencies (by age) of the period curves (Figure 1) with the birth-cohort curves (Figure 3), opposite tendencies are seen in individuals older than 29 years and in the youngest age groups (aged 0 to 14). Although there is a decreasing tendency by age in each period curve for individuals older than 29 years (Figure 1), period and cohort need to be considered to determine if AIDS incidence rates really decrease as individuals’ age increases. When birth cohort curves were plotted, an increasing trend by age could be seen in each birth cohort.

Since the cohort effect may result from lifetime experiences of the individuals born at a given point in time that have influence on the disease, the variability of past exposures in successive generations (birth cohorts) can distort the apparent associations between age and disease incidence observed at any given point in time. The health status of an individual at a point in time may be partially dependent on past exposures, such as behavioral changes that occur over time that may affect the health status of individuals [12]. For example, changes in incidence of AIDS may result from cohort effects related to greater condom use and use of disposable equipment for blood collection and sterile needles for the injecting drug users. The health status of a child at any given point in time also depends on the parents’ past exposures (i.e., behaviors that can affect their health status), since, besides the important role of the interventions in reducing perinatal transmission of AIDS, an essential factor affecting the number of children infected with HIV is the number of HIV-infected women of childbearing age. If changes in behaviors occurred in the population over time, we could expect that the successive birth cohorts change the degrees of exposure to AIDS risk factors.

In the last two periods analyzed (2000–04 and 2005–09), shown in Figure 4, a strong age effect can be observed in both sexes – that is, rates decrease with age for individuals born before 1975 and after 1990 and also increase dramatically with age for the individuals born between 1975 and 1990. Almost no cohort effect can be detected over the last 10 years analyzed (2000–09), as indicated by the practically overlapping data of the successive birth-cohort pairs for any age group (Figure 4).

The recurrent changes in tendencies over time shown by the cohort curves plotted for the entire study period, 1985–2009 (Figure 2), suggest that the risk of AIDS is also determined by period effect, although this effect has occurred in different times for men and women. For people aged more than 30 years, there is a peak in the rates in 1997 (1995–99) in each male birth cohort and in 2002 (2000–04) in each female birth cohort. Although at first glance this pattern might be considered an “age effect,” it should be noted that for each cohort older than 30 years, the maximum AIDS incidence value coincides with a single point in time (1995–99 for males and 2000–04 for females). That is, in males it corresponds to 32 (30–34) years old in the 1965 (1963–67) birth cohort, 37 (35–39) years old in the 1960 (1958–62) birth cohort, 42 (40–44) years old in the 1955 (1953–57) birth cohort, and so on. The consistent changes in curve tendency at a given time can be observed over all birth cohorts, suggesting that period effects can also explain the changes in AIDS incidence. The adjusted risk of AIDS (Figure 5C and D) shows an increasing trend by period from 1987 to 2002. The results of the period effect of AIDS, adjusted by sex and age, show a peak in the rate ratios in 2002 (Figure 5C). The results adjusted by sex and birth cohort do not show a peak over the period, although one can observe a reduction in the intensity of the risk of AIDS from 2002 (Figure 5D). Different results were found in the city of São Paulo. The authors of a study conducted in 1998–1999 detected a decreasing trend in AIDS incidence over the period [27]. Data from a study conducted in Europe also revealed that the number of diagnosed HIV infections has been holding relatively stable since 2004. The rates decreased slightly from 6.5 per 100,000 persons in 2004 to 5.7 per 100,000 individuals in 2010 [8]. Data from the Brazilian Health Ministry also show that AIDS increased gradually until 2002 and then began to decline gradually until 2007. However, in 2009 an increase of 2.9% compared to the previous year was reported [14].

In general, period effect occurs by overall changes in trends that affect the rates across birth cohort and age groups. This effect can be present if any phenomenon occurs at a specific point (or period) of time that affects the entire population (or a significant segment of the population), such as new interventions which can produce changes independently of age and cohort [12]. In the state of Rio de Janeiro, antiretroviral therapy began in 1996. In this study, changes in the AIDS incidence trend by age can be observed after 1996 (Figure 2). These changes first occurred in men (peak in 1995–99) and shortly thereafter in women (peak in 2000–04). Before these points in time (1997 for males and 2002 for females), incidence rates generally increased with age each birth cohort After this, rates by age increased only for those born between 1975 (1973–77) and 1990 (1988–92). The other birth cohorts began to undergo a decreasing tendency with age.

There is an intrinsic mathematical relationship amongst cohort, period, and age (i.e., an individual’s year of birth is calendar time minus age) [28]. One can formalize that cohort effects result from the interaction between age and calendar time, meaning that calendar time modifies the strength or the nature of an association between age and AIDS occurrence [12,29]. Age-related AIDS occurrence changes over time as a result of changing risk factors (e.g., implementation of preventive health care policies, individuals’ behavior changes). In other words, calendar time-related changes in risk factors modify the association between age and AIDS.

Period effects associated with incidence rates tend to be more prominent for diseases for which the cumulative effects of previous exposures are relatively unimportant, such as infectious diseases [12]. AIDS is an infectious chronic disease, and both cohort effects (cumulative) and period effects must be considered, since both effects can alter incidence rates simultaneously.

An important limitation of this study for the interpretation of AIDS incidence trends is the deficiency in the coverage of the epidemiologic surveillance system. The data in this study exclude not only individuals who are infected and asymptomatic but also underreporting cases. Studies on underreporting in Brazil conducted over the last decade suggest underreporting rates from 15% to 43.3% [30,31]. A recent study conducted in the state of Rio de Janeiro indicated that the greatest risk of underreporting AIDS cases occurs in women, in individuals younger than 13 years, and in older records (those registered at the beginning of the 1990s or earlier) [32].

Another factor that can cause subregistration, especially in the most recent years analyzed, is related to the missing data due to registration delay, i.e., cases of AIDS that have not been registered in the system, despite having been diagnosed [33].

Although this study focuses only on patients who developed AIDS and not in asymptomatic HIV-infected individuals, it is important to consider for the estimates of HIV-infected that the HIV incubation period, which was already long in the beginning of the epidemic, has been further increased by the introduction of combination antiretroviral treatment [33].

Recent decades have witnessed great biomedical and behavioral advances in preventing, diagnosing, and treating HIV. A transformation has been noted between 1981 when AIDS was a rapidly fatal condition to the present remarkable prolonged survival pattern. However, much remains to be done on the long road to eradication of HIV disease. Preventive health politics, like health education, must continue to play key roles in reducing AIDS risk and the impact of the epidemic on affected individuals, integrating combinations of biomedical, behavioral, and structural interventions.

## Conclusion

Age, birth cohort, and period effects all may have influenced the AIDS incidence rates over the period investigated. From 1985 to 1999, comparison of the tendencies (by age) of the period with the birth cohort revealed opposing tendencies in individuals older than 29 years and in the youngest age groups (aged 0 to 14). From 2000 to 2009, a strong age effect can be observed in both sexes. Consistent changes in period tendency curves suggest the occurrence of period effects. A reduction in the intensity of the risk of AIDS can be observed after 2000–2004.

## Competing interests

The author declares that she has no competing interests.
